# Characterization of a new semi-dominant dwarf allele of *SLR1* and its potential application in hybrid rice breeding

**DOI:** 10.1093/jxb/ery243

**Published:** 2018-06-28

**Authors:** Zhigang Wu, Ding Tang, Kai Liu, Chunbo Miao, Xiaoxuan Zhuo, Yafei Li, Xuelin Tan, Mingfa Sun, Qiong Luo, Zhukuan Cheng

**Affiliations:** 1 State Key Laboratory for Conservation and Utilization of Bio-Resources in Yunnan, Yunnan Agricultural University, Kunming, China; 2 Institute of Rice Research, Agriculture College, Yunnan Agricultural University, Kunming, China; 3 State Key Laboratory of Plant Genomics, Institute of Genetics and Developmental Biology, Chinese Academy of Sciences, Beijing, China; 4 Institute of Agricultural Science in Jiangsu Coastal Areas, Yancheng, China

**Keywords:** Giberellin, rice, semi-dominant dwarf, *SLR1*, yield

## Abstract

The widespread introduction of *semi-dwarf1* (*sd1*), also known as the ‘Green Revolution’ gene, has dramatically increased rice yield. However, the extensive use of limited sources of dwarf genes may cause ‘bottleneck’ effects in breeding new rice varieties. Alternative dwarf germplasms are quite urgent for rice breeding. Here, we characterized a new allele of the rice *Slr1-d* mutant, *Slr1-d6*, which reduced plant height by 37%, a much milder allele for dwarfism. *Slr-d6* was still responsive to gibberellin (GA) to a reduced extent. The mutation site in *Slr1-d6* was less conserved in the TVHYNP domain, leading to the specific semi-dominant dwarf phenotype. Expression of *SLR1* and five key GA biosynthetic genes was disturbed in *Slr1-d6*, and the interaction between Slr1-d6 and GID1 was decreased. In the genetic background of cultivar 9311 with *sd1* eliminated, *Slr1-d6* homozygous plants were ~70 cm tall. Moreover, *Slr1-d6* heterozygous plants were equivalent in height to the standard *sd1* semi-dwarf 9311, but with a 25% yield increase, showing its potential application in hybrid rice breeding.

## Teaser

Dwarf germplasms are very important for genetic breeding. Here, we characterize a new semi-dominant dwarf allele of *SLR1* and show its potential application in hybrid rice breeding.

## Introduction

Dwarfism is one of the most important agronomic traits in crop breeding programs. Dwarf cultivars are not only improved in lodging resistance and harvest index, but also have better responses to fertilizers (Khush, 2001). The introduction of two well-known dwarf genes, *semi-dwarf1* (*sd1*) in rice (*Oryza sativa*) and *reduced height1* (*Rht1*) in wheat (*Triticum aestivum*), to create semi-dwarf varieties has greatly increased crop yields and initiated the ‘Green Revolution’ in the 1960s (Hedden, 2003). Since then, efforts have been made by breeders to collect novel dwarf germplasms and reveal their dwarf mechanisms. More than 70 dwarf mutants have been characterized in rice so far (Liu *et al.*, 2018; https://shigen.nig.ac.jp/rice/oryzabase/).

Dwarfism in plants is mainly caused by deficiency in various endogenous hormones. Gibberellin (GA) is one of the most important phytohormones determining plant height (Wang and Li, 2005; Yamaguchi, 2008; Sun, 2011). GAs are a group of diterpenoid compounds that act as regulators in a range of growth and developmental processes in higher plants, including stem elongation, leaf differentiation, photomorphogenesis, pollen development, and flowering (Richards *et al.*, 2001; Fleet and Sun, 2005). Dwarf mutants deficient in GA biosynthesis or signaling usually exhibit a *dn*-type dwarf phenotype accompanied by deep green and rough leaves (Sakamoto *et al.*, 2004). Several genes, namely *d18* (Itoh *et al.*, 2001), *d35* (Itoh *et al.*, 2004), *sd1* (Monna *et al.*, 2002), and *eui* (Zhu *et al.*, 2006), that contribute to a defective GA biosynthetic pathway have been cloned in rice. In addition, genes encoding the GA receptor GA-INSENSITIVE DWARF1 (GID1) (Ueguchi-Tanaka *et al.*, 2005), DELLA proteins (Peng *et al.*, 1997; Silverstone *et al.*, 1998; Ikeda *et al.*, 2001), and the F-box protein GA-INSENSITIVE DWARF2 (GID2) (Gomi *et al.*, 2004) have also been isolated, and an integrated GA signaling pathway has started to emerge (Sun, 2010, 2011). DELLA protein acts as a GA signaling repressor that restrains the expression of GA-responsive genes in the absence of GA (Eckardt, 2007; Schwechheimer, 2008). When GA is present, GID1 binds to GA and subsequently interacts with DELLA proteins in the nucleus, resulting in the recognition of DELLA proteins by the SCF^GID2/SLY1^ complex, degradation of DELLA proteins via the 26S proteasome pathway, and consequently activation of the expression of GA-responsive genes (Sun and Gubler, 2004). Despite the fact that numerous dwarf or semi-dwarf mutants have been reported, only *sd1* and its alleles act as useful dwarf sources and thus have been widely used for rice breeding (Asano *et al.*, 2007).

Intervarietal hybrid rice yields 10–20% more than inbred varieties, and it covers >50% of the total rice-planting area in China (Cheng *et al.*, 2007). Two-line intersubspecific (*indica*/*japonica*) hybrids could further increase yield potential, exceeding the yield plateau reached by intervarietal hybrid rice. However, in hybrid breeding programs, both male and female parents must carry the same recessive dwarf gene *sd1* to solve the problem of higher plant stature due to heterosis, which is time consuming and labor intensive. Moreover, the extensive use of limited dwarf sources may cause ‘bottleneck effects’ in the genetic background when breeding new varieties, which would cause genetic vulnerability to pests or diseases (Kikuchi and Futsuhara, 1997). If one parent has a dominant dwarf gene, there will be no restriction on whether or not the other parent has a recessive dwarf gene, which would greatly expand the genetic backgrounds that can be selected for both parents and save time and labor costs in breeding high yield rice hybrids. Thus, the identification of dominant dwarf germplasms is urgent for hybrid rice breeding. However, there have only been a few dominant or semi-dominant dwarf mutants, including *D53*, *Dx*, *D-h*, *LBD4*, *Sdd*(*t*), *Slr1-d*, *Ssil*, and *Tid1*, characterized in rice so far (Wei *et al.*, 2006; Liu *et al.*, 2008; Qin *et al.*, 2008; Asano *et al.*, 2009; Miura *et al.*, 2009; Sunohara *et al.*, 2009; Hirano *et al.*, 2010; Liang *et al.*, 2011; Piao *et al.*, 2014; Zhang *et al.*, 2016). In addition, these dwarf mutants are not available for practical breeding due to their unfavorable phenotypes, such as severe dwarfism, low fertility, and short grains. In rice, there are currently no dominant dwarfing sources with high breeding value that are being used in practical production.

Five alleles of *Slr1-d* mutants have been previously reported in rice. They showed ~50–70% reduction in plant height when compared with their wild types (Asano *et al.*, 2009; Hirano *et al.*, 2010; Zhang *et al.*, 2016). In the present study, we characterized a new mutant allele of *Slr1-d*, *Slr1-d6*. The *Slr1-d6* mutant reduces plant height by 37%, which is much milder dwarfism than previously reported alleles. Map-based cloning revealed that the mutation site in *Slr1-d6* is less conserved in the TVHYNP domain of SLR1, which is thought to be the reason for the milder dwarf phenotype in *Slr1-d6*. Expression of *SLR1* and five key GA biosynthetic genes is disturbed in the *Slr1-d6* mutant. The S97L substitution in SLR1 leads to a decreased interaction with GID1. In the genetic background of cultivar 9311 with *sd1* eliminated, *Slr1-d6* homozygous plants showed suitable plant height and decent seed setting for sterile line production. Moreover, *Slr1-d6* heterozygous plants also showed significant yield potential compared with normal 9311 plants.

## Materials and methods

### Plant materials and growth conditions

The semi-dominant dwarf mutant *Slr1-d6* is a spontaneous mutant identified from the *indica* rice (*Oryza sativa* L.) cultivar Zhongxian 3037 (ZX3037). The F_2_ mapping populations were generated by crossing *Slr1-d6*^*+/*−^ plants with the *japonica* variety Nipponbare. The *indica* variety Nanjing 6, with no dwarf genes, was used as one donor parent. Four 9311 plant lines with different genotypes of dwarf genes, namely *SD1SD1*/*SLR1SLR1*, *sd1sd1***/***SLR1SLR1*, *SD1SD1*/*Slr1-d6SLR1*, and *SD1SD1*/*Slr1-d6Slr1-d6*, were generated with the following steps: first, *Slr1-d6*^*+/*−^ plants were crossed with Nanjing 6, and *SD1SD1*/*Slr1-d6Slr1-d6* plants were chosen at the segregation generation; secondly, *SD1SD1*/*Slr1-d6Slr1-d6* plants were crossed with 9311, and then *SD1SD1*/*Slr1-d6Slr1-d6* plants were chosen at the segregation generation; and, thirdly, *SD1SD1*/*Slr1-d6Slr1-d6* plants were backcrossed six times with 9311 followed by a final selfing generation. All plant materials were cultivated in paddy fields in Beijing or Yangzhou in the summer and in Hainan in the winter, with spacing of 13.3 cm between plants within each row and 25 cm between rows, under normal rice production practices.

### Culm anatomical observation

At the filling stage, the second internodes under the panicle of wild-type ZX3037 and *Slr1-d6* plants were fixed in FAA solution (5% formaldehyde, 5% glacial acetic acid, and 63% ethanol) for 2 d at 4 °C after they were vacuum pumped for 30 min. After dehydration in a graded ethanol series (30–50–70–85–90–100%; 30 min per step), the samples were infiltrated and embedded in Technovit 7100 resin (RM2265, Germany). Transverse and longitudinal sections (4 µm thick) were cut with a Leica (Wetzlar, Germany) microtome. These sections were stained with 0.25% toluidine blue and then observed under a light microscope (DM500, Leica, Germany).

### GA induction of shoot elongation

Seed glumes were removed and sterilized with 75% ethanol for 5 min, then sterilized with 25% NaClO for 40 min, and finally washed five times with sterile distilled water and put onto sterilized filter paper to remove residual moisture. The seeds were cultivated on 1/2 Murashige and Skoog (MS) solid medium containing different concentrations of GA_3_ and grown at 28 °C with 12 h of light from fluorescent lights. Ten days later, the length of the second leaf sheath of each plant was measured.

### Map-based cloning and sequence analysis of *Slr1-d6*

A total of 523 plants with a severe dwarf phenotype segregated from the F_2_ population from a cross between *Slr1-d6* and Nipponbare were selected for isolating the mutated gene. Sequence-tagged site (STS) markers were developed according to sequence differences between the *japonica* variety Nipponbare and the *indica* variety 9311, according to the data published on the NCBI website (http://www.ncbi.nlm.nih.gov). Orthologs of rice DELLA protein, SLR1, were downloaded from the NCBI website by homology blasting. Multiple sequence alignment was conducted using the online software Bioinformatics Toolkit (https://toolkit.tuebingen.mpg.de/#/).

### Functional complementation test

The complementary plasmid was constructed by cloning a 6.06 kb genomic DNA fragment, containing 2541 bp promotor sequences, the entire 1878 bp coding region of *Slr1-d6*, and 1669 bp downstream sequences, into the pCAMBIA1300 vector. The control plasmid contained a 2541 bp promotor sequence, the entire 1878 bp coding region of *SLR1*, and 1669 bp downstream sequences. Transformation of the embryonic callus of ZX3037 was conducted using the recombinant plasmids constructed above. Genotypes of the transgenic plants were identified by sequencing.

### Quantitative real-time PCR (qRT-PCR) for transcript expression assay

Total RNA was extracted from root, internode, young leaf, leaf sheath, and panicle of ZX3037 and *Slr1-d6* at the elongation stage using the classical TRIZOL RNA isolation protocol (Chomczynski and Mackey, 1995); RNA was further purified with DNase I. The mRNAs were reverse transcribed into cDNA with oligo(dT)_18_ primer. Real-time PCR was performed using the Bio-Rad CFX96 real-time PCR instrument and EvaGreen (Biotium, http://www.biotium.com/) with gene-specific primer pairs (SLR1-RT-F/R, OsCPS1-RT-F/R, OsKS1-RT-F/R, OsKAO-RT-F/R, OsGA3ox2-RT-F/R, and SD1-RT-F/R) and an internal control primer OsActin-RT-F/R. The results were analyzed using OPTICONMONITOR 3.1 (Bio-Rad, USA). Each experiment had three replicates.

### Yeast two-hybrid assay

The Matchmaker Two-Hybrid System (Clontech, USA) was used for the yeast two-hybrid assay. pGADT7-SLR1 and pGADT7-Slr1-d6 served as the prey, and pGBKT7-GID1 as the bait. Plate assays (–His) and β-galactosidase (β-gal) liquid assays were conducted according to the manufacturer’s protocol (Clontech) with 10^–4^ M GA_3_ added or not added (control). The yeast strain Y2H gold was used for growth tests on –His plates. Serial 1:10 dilutions were prepared in ddH_2_O, and 10 µl of each dilution was used per spot; Y187 was used to detect β-gal activity by liquid assay.

### Measurement and statistical analysis of yield-associated agronomic traits

All of the following recordings and measurements were carried out at the maturity stage. Effective tillers of 20 and 100 plants of each sample were evaluated in Beijing and Yangzhou, respectively. The length from the ground surface to the top of the highest panicle was measured as the plant height of rice, and 20 and 50 plants for each sample were investigated in Beijing and Yangzhou, respectively. Yield per plant was represented by the average product of 10 plants that were randomly selected in the middle field. Spikelets per panicle were calculated by: total spikelets of one plant/effective tiller number, with 10 replications. Seed setting (%) was calculated by: filled grains per panicle/spikelets per panicle×100%. Kilo-grain weight was measured for each sample with five replications. All analyses were conducted using the statistical data analyses software SPSS.

## Results

### Identification and characterization of a novel semi-dominant dwarf mutant in rice

**Fig. 1.**
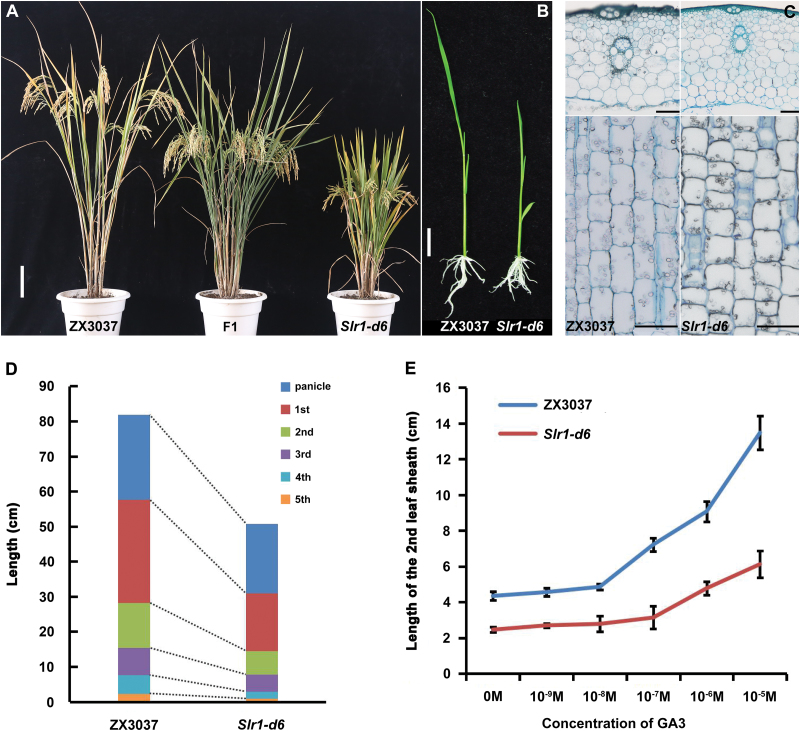
Characterization of the phenotype and response to exogenous GA_3_ treatment of the semi-dominant dwarf mutant *Slr1-d6.* (A) Gross morphology of ZX3037 (left), F_1_ plant between ZX3037 and *Slr1-d6* (middle), and homozygous mutant *Slr1-d6* (right). Scale bar=10 cm. (B) Morphology of 10-day-old seedlings of ZX3037 (left) and *Slr1-d6* (right). Scale bar=2 cm. (C) Transverse and longitudinal sections of the penultimate internode from ZX3037 (left) and *Slr1-d6* (right). Scale bars=100 µm. (D) Panicle and internode length comparison between ZX3037 and *Slr1-d6*. (E) Elongation of the second leaf sheath of *Slr1-d6* in response to exogenous treatment with different concentrations of GA_3_. ZX3037 was used as a control. Data are means ±SD; *n*=10.

A rice spontaneous dwarf mutant was isolated from ZX3037. The mutant plant showed a 37% reduction in plant height compared with the normal ZX3037 (Supplementary Table S1 at *JXB* online), with wide and shortened dark green leaf blades (Fig. 1A). When the mutant plant was crossed with the normal ZX3037, the F_1_ plants showed intermediate plant height relative to both parents (Fig. 1A). Ten-day-old seedlings of *Slr1-d6* were also shorter than those of ZX3037, but had more developed roots (Fig. 1B). Transverse and longitudinal sections of the penultimate internode of the dwarf mutant showed that the cell length was significantly reduced and the internode was obviously thickened with increased cell layers (Fig. 1C). In the subsequent F_2_ population, the segregation ratio of dwarf plants (including semi-dwarf phenotype like the F_1_ plants) to normal plants was 73:25, which is consistent with the expected 3:1 segregation ratio of a single dominant gene. In comparison with ZX3037, the lengths of different internodes of the dwarf mutant were all reduced (Fig. 1D; Supplementary Table S1), characteristic of the *dn*-type rice dwarf mutants (Takeda, 1977). These results indicated that this dwarf mutant was a semi-dominant dwarf mutant belonging to the *dn*-type rice dwarf mutants, and that cell length reduction may be the immediate cause of the shortened culm length in the dwarf mutant plant.

Defects in GA biosynthesis and/or perception are the major determinants of plant height, despite various causes of dwarfism in plants. In the present study, the response of the dwarf mutant to exogenous GA_3_ was examined using a shoot elongation test. Elongation of the second leaf sheath of the wild type was clear with the application of 10^–8^–10^–7^ M GA_3_, while such effects were not observed in the mutant until 10^–7^–10^–6^ M GA_3_ was applied (Fig. 1E). When treated with 10^–5^ M GA_3_, the length of the second leaf sheath in ZX3037 was elongated up to ~13.5 cm, whereas that in the mutant was only elongated to ~6 cm (Fig. 1E). These results suggest that the dwarf mutant is responsive to GA, although to a reduced extent.

### Map-based cloning and molecular analysis of *Slr1-d6*

**Fig. 2.**
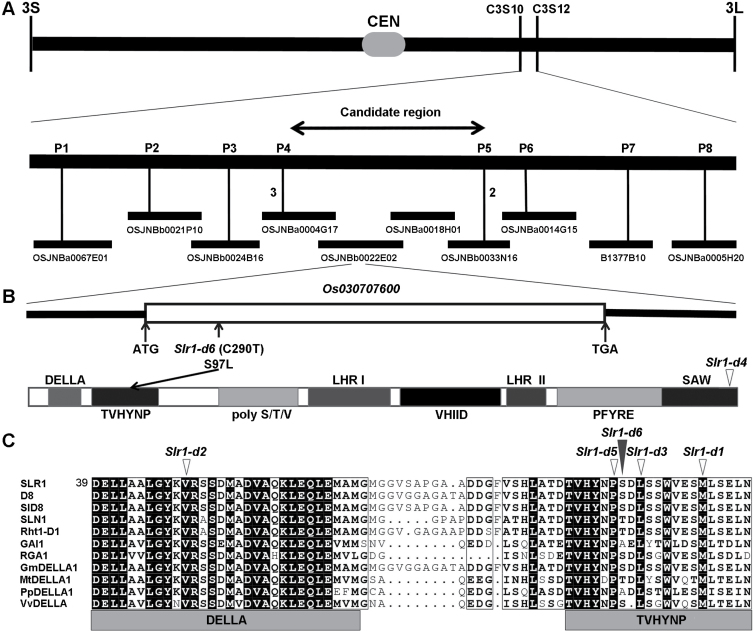
Map-based cloning of *Slr1-d6*. (A) The *Slr1-d6* locus was mapped to a 356 kb candidate region on the long arm of chromosome 3. The vertical bars represent molecular markers, and the adjacent numbers indicate recombinant plants. (B) Gene structure and mutation site on the candidate gene *Os030707600*. White boxes with black frames indicate exons, and black bold lines on both sides represent the 5'- and 3'-untranslated regions. Boxes with different gray levels indicate different motifs deduced in the rice DELLA protein, SLR1. (C) Multiple sequence alignment of the conserved N-terminal domains of different DELLA proteins. DELLA proteins from rice (SLR1), maize (D8), sorghum (SlD8), barley (SLN1), wheat (Rht1-D1), Arabidopsis (GAI1 and RGA1), soybean (GmDELLA1), alfalfa (MtDELLA1), pear (PpDELLA1), and grape (VvDELLA). The black triangle indicates the mutation site of *Slr1-d6*. The white triangles indicate the mutation sites of other *Slr1-d* allele mutants.

To isolate the mutated gene in *Slr1-d6* that controls the dwarf phenotype, map-based cloning was carried out using the F_2_ population generated by crossing the dwarf mutant with Nipponbare. In total, 523 plants with severe dwarf phenotypes that segregated from the F_2_ population were selected and used for gene mapping. Preliminarily, the mutated gene locus was located between two STS markers, C3S10 and C3S12, on the long arm of chromosome 3 (Fig. 2A). Using adjacent insertion/dletion (InDel) markers (Supplementary Table S2), we further narrowed its locus to a 356 kb candidate region that contains 52 predicted genes (Fig. 2A). Scanning this candidate region, we found that the gene *Os03g0707600* that encodes the sole rice DELLA protein, SLR1, is located within this region. Five dominant dwarf mutant alleles of SLR1, including *Slr1-d1*-*d5*, showing dominant dwarf and delayed GA response or GA-insensitive phenotypes, have been reported in rice (Asano *et al.*, 2009; Hirano *et al.*, 2010; Zhang *et al.*, 2016). Thus, *Os03g0707600* was considered as the most likely candidate gene and was sequenced. Sequence analysis revealed that a single base transition, C290T, occurred in *SLR1* in the dwarf mutant, leading to substitution of the 97th amino acid serine (S) of SLR1 with leucine (L) (Fig. 2B). Multiple sequence alignment of the conserved N-terminal domains of DELLA proteins showed that, unlike previously reported *Slr1-d* alleles, the substituted amino acid residue site of SLR1 in the dwarf mutant was less conserved (Fig. 2C). We named the semi-dominant dwarf mutant *Slr1-d6* and suspected that the single base transition C290T of *SLR1* conferred the dwarf phenotypes described above.

To confirm the above speculation, transgenic plants expressing *SLR1* (ZX3037^*SLR1*^) and *Slr1-d6* (ZX3037^*Slr1-d6*^) under the control of their own promoters were generated, and 19 and 14 transgenic lines were obtained, respectively. Ten-day-old seedlings and mature plants of transgenic lines expressing *Slr1-d6* had a dwarf phenotype, while transgenic lines expressing *SLR1* were not obviously different from the wild-type ZX3037 (Supplementary Fig. S1). These results further confirmed that the gain-of-function mutant of the rice DELLA protein SLR1 caused the semi-dominant dwarf phenotypes described in *Slr1-d6*.

### Expression of *SLR1* and five key GA biosynthetic genes was disturbed in *Slr1-d6*

**Fig. 3.**
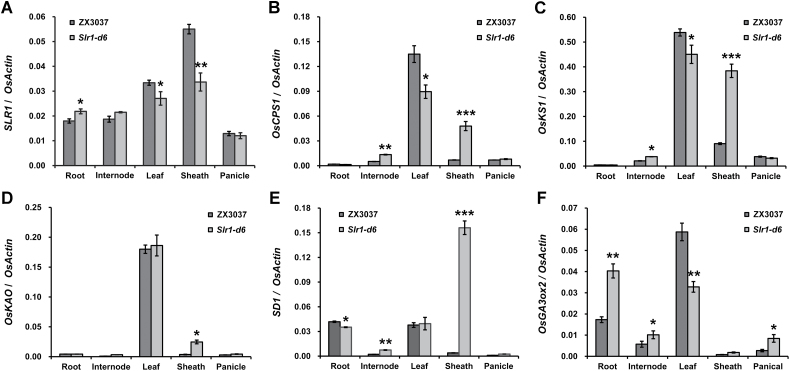
Expression patterns of *SLR1* and five key GA biosynthetic genes in *Slr1-d6.* * represents a significance level of *P*<0.05; ** represents a significance level of *P*<0.01; *** represents a significance level of *P*<0.005. Error bars are the mean ±SD (*n*=3).

DELLA protein is one of the key components of the GA signaling pathway and acts as a suppressor of GA responses. To reveal the dwarfism mechanism of *Slr1-d6* at the transcription level, we first detected the expression pattern of *SLR1* in both the mutant and its wild type by qRT-PCR at the jointing–booting stage. In the wild-type ZX3037 plants, *SLR1* was expressed in all five detected tissues, namely root, internode, leaf, sheath, and panicle, of which sheath showed the highest expression level (Fig. 3A). In the *Slr1-d6* plants, transcripts of the mutated DELLA-encoding gene were also detected in all five tissues by gene-specific qRT-PCR primers of *SLR1* (Supplementary Table S3). However, its expression was significantly down-regulated in leaf and sheath, and conversely up-regulated in root (Fig. 3A). These results seem to conflict: *Slr1-d6* showed reduced plant height and sheath length while the DELLA-encoding gene had a lower expression level than the wild type in leaf and sheath.

Numerous studies have shown that DELLA protein also plays a role in maintaining GA homeostasis by feedback regulation of the GA biosynthesis pathway (Sun and Gubler, 2004; Yamaguchi, 2008). Thus we further detected the expression patterns of five key GA biosynthetic genes, *OsCPS1*, *OsKS1*, *OsKAO*, *SD1* (*OsGA20ox2*), and *OsGA3ox2*, in *Slr1-d6* at the same stage by gene-specific primers (Supplementary Table S3). *OsCPS1* and *OsKS1* encode enzymes that catalyze early steps in the conversion of GGDP to *ent*-kaurene in the plastids; *OsKAO* encodes an enzyme that catalyzes the conversion of *ent*-kaurene acid to GA_12_ in the endoplasmic reticulum; while *SD1* and *OsGA3ox2* encode enzymes that catalyze late steps in the synthesis of bioactive GAs in the cytoplasm (Yamaguchi, 2008). In the wild-type ZX3037 plants, *OsCPS1*, *OsKS1*, *OsKAO*, and *OsGA3ox2* are all mainly expressed in young leaves (Fig. 3B–D, F), but not *SD1* which is mainly expressed in both root and young leaves (Fig. 3E). In the *Slr1-d6* plants, the expression levels of *OsCPS1*, *OsKS1*, and *OsGA3ox2* were significantly down-regulated in young leaves (Fig. 3B, C, F), and, conversely, the expression levels of *OsCPS1*, *OsKS1*, and *SD1* in internode and sheath, *OsKAO* in sheath, and *OsGA3ox2* in root and internode were significantly up-regulated (Fig. 3B–F). These results suggest that the expression patterns of the five key GA biosynthetic genes were disturbed in *Slr1-d6*.

### Interaction between Slr1-d6 and GID1

**Fig. 4.**
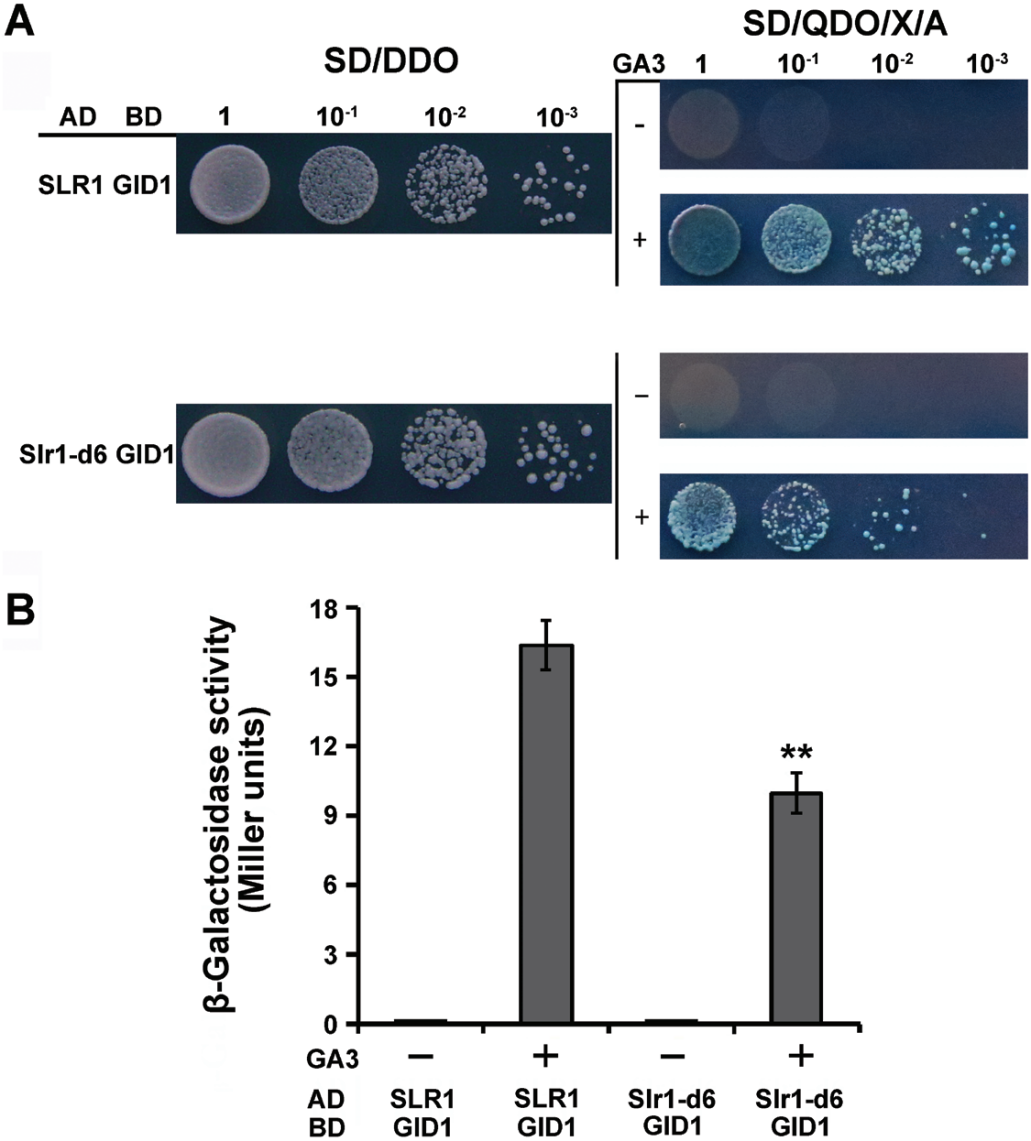
Slr1-d6 shows decreased interaction ability with GID1. (A) Yeast two-hybrid tests between Slr1-d6 and GID1. Growth of yeast strain Y2Hgold on a –His plate with 10^–4^ M GA_3_ (+) or without (–). (B) Interaction between Slr1-d6 and GID1 in an *in vitro* β-galactosidase activity tested in a liquid assay using yeast strain Y187 with (+) or without (–) 10^–4^ M GA_3_. Error bars are the mean ±SD (*n*=3).

DELLA protein interacted with the GA receptor, GID1, in a GA-dependent manner, which is the key step in GA signaling and is necessary for subsequent degradation of DELLA proteins (Gomi and Matsuoka, 2003; Eckardt, 2007; Sun, 2010; Davière and Achard, 2013). The interaction abilities of Slr1-d mutant proteins with GID1 were reduced in *Slr1-d* mutants, which caused their dwarf phenotypes (Asano *et al.*, 2009). A yeast two-hybrid assay was performed to test whether the interaction between Slr1-d6 and GID1 was affected in the *Slr1-d6* mutants. Both yeast cells, those expressing SLR1 and GID1 and those expressing Slr1-d6 and GID1, grew on –His plates in the presence of GA_3_, whereas none of the yeast cells grew on –His plates without GA_3_ (Fig. 4A). However, yeast cells expressing Slr1-d6 and GID1 grew much more slowly and less than yeast cells expressing SLR1 and GID1; both types of cells only had dispersed and small yeast colonies on –His plates with GA_3_ (Fig. 4A). These results suggest that Slr1-d6 could interact with GID1 in a GA-dependent manner just like the wild-type SLR1 and other Slr1-d proteins, but the interaction ability between Slr1-d6 and GID1 was affected.

To confirm further the above speculation, β-galactosidase activity was measured with a liquid assay using yeast strain Y187, with or without 10^–4^ M GA_3_ treatment. The β-gal activity of yeast strain Y187 expressing Slr1-d6 and GID1 was significantly lower than that of yeast strain Y187 expressing SLR1 and GID1 in the presence of GA_3_, and no β-galactosidase activity was detected in either strain in the absence of GA_3_ (Fig. 4B). These results further confirmed that the interaction between Slr1-d6 and GID1 is in a GA-dependent manner and their interaction ability is decreased.

### Yield trait performance of *Slr1-d6*

**Fig. 5.**
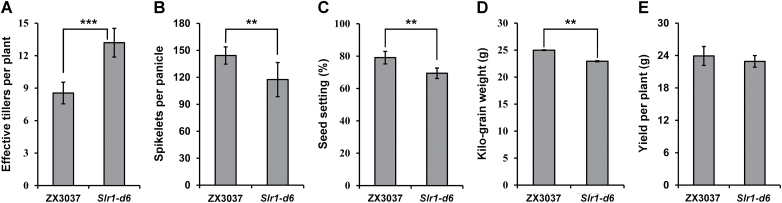
Yield trait performance of *Slr1-d6*. Sample size: *n*=10 for spikelets per panicle, seed setting, and yield per plant; *n*=20 for effective tillers per plant; *n*=5 for kilo-grain weight. Error bars are the mean ±SD. ** represents a highly significant difference (*P*<0.01), and *** represents an extremely significant difference (*P*<0.005) by *t*-test.

*Slr1-d* mutants showed 50–70% reduction in plant height in previous studies (Asano *et al.*, 2009; Hirano *et al.*, 2010; Zhang *et al.*, 2016). In the present study, *Slr1-d6* had a 37% reduction in plant height. The *Slr-d6* mutant thus has much milder dwarfism than previously reported alleles, which stimulated our interest in investigating its potential application in rice breeding. Statistical analyses of yield traits, including effective tillers per plant, spikelets per panicle, seed setting, kilo-grain weight, and yield per plant of *Slr-d6* and ZX3037, were carried out at the mature stage. Compared with ZX3037, the number of effective tillers per plant of *Slr-d6* was significantly increased (Fig. 5A), while the spikelets per panicle, seed setting, and kilo-grain weight of *Slr-d6* were significantly decreased (Fig. 5B–D). Surprisingly, the yield per plant of *Slr-d6* showed no significant difference from ZX3037 (Fig. 5E). These results suggest that the *Slr1-d6* mutant may have some negative influences on agronomy traits, but its yield would not be affected for the compensation effects by increased tillers, suggesting that *Slr1-d6* may have potential application value.

### Comparison of dwarfing ability between *Slr1-d6* and *sd1*

**Fig. 6.**
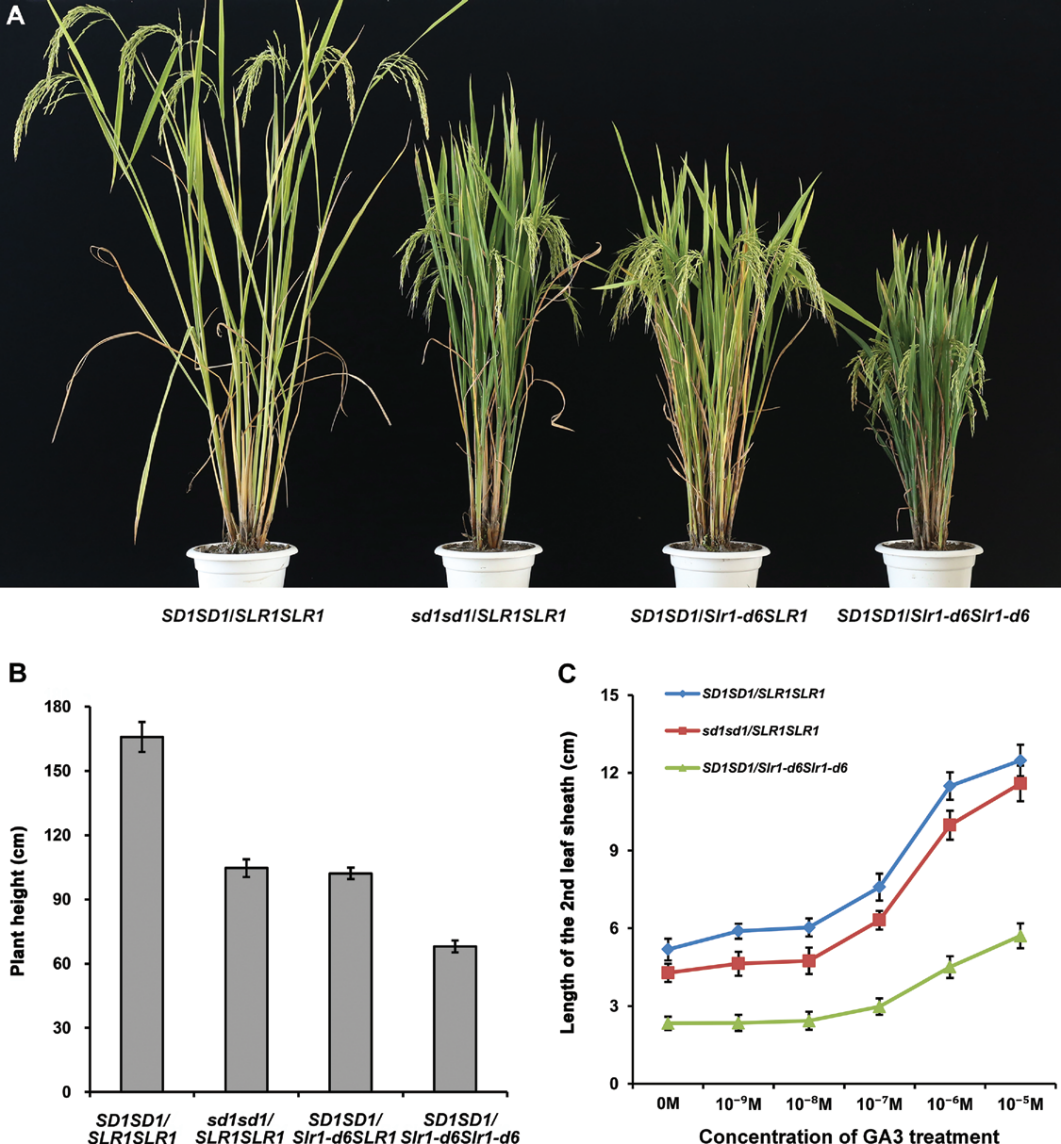
Dwarfing ability comparison between *Slr1-d6* and *sd1*. (A) Gross morphology of 9311 plants with different genotypes of dwarf genes at the mature stage. (B) Plant height in the 9311 genetic background with different dwarf genes. Plant heights were measured at the mature stage. Data are means ±SD; *n*=50. (C) Elongation of the second leaf sheath of different 9311 lines in response to GA_3_ treatment. Data are means ±SD; *n*=10.

The *Slr1-d6* mutant in the ZX3037 genetic background showed milder dwarfism, which may be useful in rice dwarf breeding. However, the cultivar ZX3037 intrinsically has the semi-dwarf gene *sd1*, making it difficult to assess the dwarfism effects of *Slr1-d6* accurately in the ZX3037 genetic background. To exclude the influence of *sd1* and accurately evaluate the dwarfing effects of *Slr1-d6*, we constructed plant lines with different genotypes of dwarf genes in the same genetic background of cultivar 9311 (see the Materials and Methods for details). The line without any dwarf genes (*SD1SD1/SLR1SLR1*) had the highest plant height; the line with the green revolution gene *sd1* (*sd1sd1/SLR1SLR1*), the wild-type cultivar 9311, had a semi-dwarf phenotype accompanied by dark-green and erect leaves; the line with heterozygous genotype of *Slr1-d6* (*SD1SD1/Slr1-d6SLR1*) was similar to normal 9311 in plant height but matured earlier; the line with the homozygous genotype of *Slr1-d6* (*SD1SD1/Slr1-d6Slr1-d6*) had the lowest plant height, with leaves that were shorter and greener than the wild type 9311 (Fig. 6A). Further measurement of plant height revealed that the plant heights of *SD1SD1/SLR1SLR1*, *sd1sd1/SLR1SLR1*, *SD1SD1/Slr1-d6SLR1*, and *SD1SD1/Slr1-d6Slr1-d6* were 165.85 ± 7.07, 104.60 ± 4.19, 102.18 ± 2.62, and 68.02 ± 2.69 cm, respectively (Fig. 6B; Supplementary Table S4). The *Slr1-d6* homozygous plants were shorter than the *sd1* homozygous plants in the same genetic background of 9311, suggesting that the dwarfing ability of *Slr1-d6* is stronger than that of *sd1*. The height of *Slr1-d6* homozygous plants was close to 70 cm, which is not too short for sterile line production. In addition, the plant height of *Slr1-d6* heterozygotes was equivalent to the normal semi-dwarf height of 9311 plants carrying *sd1*. These results suggest that *Slr1-d6* may have potential application value in rice hybrid breeding.

In addition, the responses of *Slr1-d6* and *sd1* mutants to exogenous GA_3_ treatment were also tested in the same genetic background of 9311. Lines *SD1SD1/SLR1SLR1* and *sd1sd1/SLR1SLR1* had the same response tendency to exogenous GA_3_ treatment: obvious elongation of the second leaf sheath was observed with the application of 10^–8^–10^–7^ M GA_3_ (Fig. 6C). Similar to *Slr1-d6*, this response of the *SD1SD1/Slr1-d6Slr1-d6* plant line was not observed until the application of 10^–7^–10^–6^ M GA_3_ (Fig. 6C). These results further confirmed that the *Slr1-d6* mutant is responsive to GA, although to a reduced extent, which is not affected by the presence or absence of *sd1*.

### Yield trait performance of *Slr1-d6* in the genetic background of 9311

**Fig. 7.**
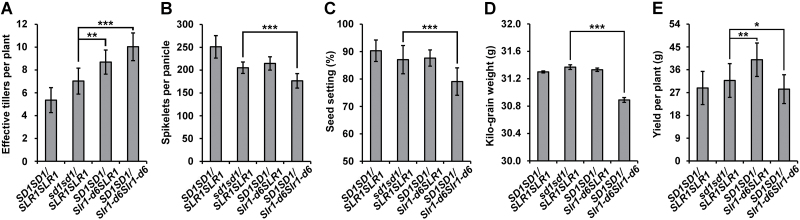
Yield trait performance of *Slr1-d6* in the genetic background of 9311. (A) Number of effective tillers per plant, means ±SD; *n*=100. (B) Spikelets per panicle, means ±SD; *n*=10. (C) Seed setting, means ±SD; *n*=10. (D) Kilo-grain weight, means ±SD; *n*=5. (E) Yield per plant, means ±SD; *n*=10. * represents a significant difference (*P*<0.05), ** represents a highly significant difference (*P*<0.01) and *** represents an extremely significant difference (*P*<0.005) by *t*-test.

To evaluate further the practical application value of *Slr1-d6* in rice hybrid breeding, a comparison of yield traits between *Slr1-d6* and *sd1* was also carried out in the same genetic background of 9311 at the mature stage (Supplementary Table S4). Effective tillers per plant of *SD1SD1*/*Slr1-d6Slr1-d6* and *SD1SD1*/*Slr1-d6SLR1* plants were 10.04 ± 1.22 and 8.70 ± 1.06, respectively, both significantly higher than 7.03 ± 1.09 in *sd1sd1*/*SLR1SLR1* plants (Fig. 7A). Spikelets per plant of *sd1sd1*/*SLR1SLR1* and *SD1SD1*/*Slr1-d6SLR1* plants were 205.30 ± 12.66 and 214.80 ± 14.59, respectively, significantly higher than 176.50 ± 15.91 in *SD1SD1*/*Slr1-d6Slr1-d6* plants (Fig. 7B). The seed setting rates of *SD1SD1*/*Slr1-d6Slr1-d6* and *SD1SD1*/*Slr1-d6SLR1* plants were 87.06 ± 5.17% and 87.65 ± 2.96%, respectively, significantly higher than 79.08 ± 5.02% in *SD1SD1*/*Slr1-d6Slr1-d6* plants (Fig. 7C). Kilo-grain weights of *sd1sd1*/*SLR1SLR1* and *SD1SD1*/*Slr1-d6SLR1* plants were 31.37 ± 0.03 g and 31.33 ± 0.02 g, respectively, higher than 30.89 ± 0.04 g in the *SD1SD1*/*Slr1-d6Slr1-d6* plants (Fig. 7D). Yield per plant of *sd1sd1*/*SLR1SLR1* plants was 31.75 ± 6.67 g, and in *SD1SD1*/*Slr1-d6Slr1-d6* plants it was 28.35 ± 5.70 g. In contrast, the yield per plant of *SD1SD1*/*Slr1-d6SLR1* plants was as high as 39.95 ± 6.59 g, a significant increase of 25% compared with *sd1sd1*/*SLR1SLR1* plants (Fig. 7E; Supplementary Table S5).

The number of effective tillers per plant increased dramatically, but spikelets per panicle, seed setting rate, kilo-grain weight, yield per plant, and yield per square meter of *Slr1-d6* homozygous plants were all significantly lower than in *sd1* homozygous plants. The seed setting of *Slr1-d6* homozygous plants is close to 80%, which is sufficient for F_1_ hybrid seed production. In addition, *Slr1-d6* heterozygous plants showed great yield-increasing potential when compared with *sd1* homozygous plants. These results suggest that *Slr1-d6* is a valuable dominant dwarf allele that has potential application value in rice hybrid breeding.

## Discussion

Currently, >70 rice dwarf mutants and 24 wheat dwarfing genes have been reported (Tian *et al.*, 2017; Liu *et al.*, 2018). However, most of these dwarf mutants are accompanied by unfavorable phenotypes such as severe dwarfism, low fertility, and short grains; thus *sd1* and *Rht1* are still the dwarf sources predominantly used to produce modern semi-dwarf varieties in rice and wheat, respectively (Hedden, 2003; Asano *et al.*, 2007). Both *sd1* and *Rht1* are related to GA. *SD1* encodes the GA biosynthetic enzyme, GA20 oxidase 2, catalyzing late steps of gibberellin biosynthesis, and its mutation reduces the endogenous GA level, causing the semi-dwarf phenotypes in *sd1* mutants (Sasaki *et al.*, 2002; Spielmeyer *et al.*, 2002). In contrast, the *RHT1* encodes a GA signaling repressor DELLA protein; deletion in the N-terminal region constitutively suppresses GA signaling and consequently results in a dominant, semi-dwarf phenotype (Peng *et al.*, 1999). Both cases highlight the pivotal role of GA in regulating plant height, making the GA pathway a prime target for generating useful dwarf sources for crop breeding (Hedden, 2003).

In this study, we isolated and characterized a new allele of rice *Slr1-d* mutants, *Slr1-d6*. Similar to the five previously reported alleles of *Slr1-d* mutants, *Slr1-d6* had wide, dark-green leaf blades, with reduced response to exogenous GA treatment, and reduced elongation of all internodes. *Slr-d6* is inherited in a semi-dominant dwarf manner. Based on anatomical observations of the culm, we concluded that cell length reduction was the immediate reason for plant height reduction in *Slr1-d6*. However, the *Slr1-d1*, *-d2*, *-d3*, *-d4*, and *-d5* mutants had ~50–70% reduction in plant height (Asano *et al.*, 2009; Hirano *et al.*, 2010; Zhang *et al.*, 2016), while *Slr-d6* mutant plants only led to a 37% reduction in plant height, which is much milder dwarfism than for previously reported alleles. *Slr1-d6* has a 1 bp substitution resulting in the amino acid substitution S97L in the conserved TVHYNP motif of SLR1. Based on multiple sequence alignment of the conserved DELL1/TVHYNP domain of DELLA protein orthologs, we found that the amino acid of the mutation site in *Slr1-d6* is less conserved than other alleles, which might be the reason for milder dwarfism in *Slr1-d6*.

DELLA protein is one of the key components of the GA signaling pathway and acts as a suppressor of GA responses. The expression of *SLR1* in the root of *Slr1-d6* was up-regulated, which is consistent with the dwarf phenotype. Conversely, the expression of *SLR1* in both the leaf and internode of *Slr1-d6* was down-regulated, which is not consistent with the dwarf phenotype. This conflicting result suggests that the dwarf mechanism hidden in *Slr1-d6* could not be explained by its mutated DELLA-encoding gene at the transcriptional level. DELLA protein also plays a role in maintaining GA homeostasis by feedback regulation of the GA biosynthesis pathway (Sun and Gubler, 2004; Yamaguchi, 2008). When a loss-of-function mutation occurs in DELLA protein, the transcript levels of GA20ox and/or GA3ox genes are lower than in the wild type. Conversely, gain-of-function mutations in DELLA protein often result in up-regulation of *GA20ox* and *GA3ox* gene expression. *Slr1-d6* is a gain-of-function mutant of rice DELLA protein. The expression of *SD1* in the internode and sheath and *OsGA3ox2* in the root and internode of *Slr1-d6* was up-regulated, consistent with previous studies. In contrast, *SD1* in the root and *OsGA3ox2* in the leaf of *Slr1-d6* were down-regulated. There are two possible reasons to explain this paradoxical phenomenon: first, the feedback regulation of the GA biosynthesis pathway mediated by gain-of-function mutation in DELLA protein has tissue specificity, which mainly happened in the rapidly elongated tissues such as internode and sheath in rice at the jointing–booting stage; secondly, the changed expression of one GA biosynthetic gene in one tissue may also have a feedback regulation on its expression in the other tissues to maintain GA homeostasis. Similar to *SD1* and *OsGA3ox2*, the expression of *OsCPS1*, *OsKS1*, and *OsKAO* was mainly up-regulated in internode and/or sheath, and down-regulated in leaf. This is consistent with the current opinion that downstream steps usually regulate feedback on upstream steps in the same biosynthesis pathway. Previous studies have mainly focused on one tissue to illustrate the feedback regulation of DELLA protein on the GA biosynthesis pathway. Whether the feedback regulation of DELLA protein on the GA biosynthesis pathway has tissue specificity still needs to be confirmed.

DELLA protein may interact with the GA receptor, GID1, in a GA-dependent manner, which guides its degradation via the 26S proteasome pathway (Sun and Gubler, 2004). Based on a yeast two-hybrid assay and a β-gal activity test, we found that the mutated SLR1 protein, Slr1-d6, still interacted with GID1 in a GA-dependent manner, but with decreased interaction ability. Previous studies have also revealed that DELLA proteins interact with GID1 via the N-terminal DELLA/TYHYNP domain of DELLA protein (Ueguchi-Tanaka *et al.*, 2007; Willige *et al.*, 2007), especially the valine and proline residues of the TVHYNP motif that directly binds to GID1 (Murase *et al.*, 2008). The S97L substitution in Slr1-d6 is adjacent to the important proline residues of the TVHYNP motif; thus we speculate that the 97th serine residue of the TVHYNP motif also plays a role in the interaction between SLR1 and GID1. Taken together, the semi-dominant dwarf phenotypes of *Slr1-d6* might be caused by inefficient degradation of the mutated DELLA protein, Slr1-d6.

Dominant dwarf germplasms possess special advantages in rice hybrid breeding programs. When one parent carries a dominant dwarf gene, it does not matter whether the other parent has a recessive dwarf gene, meaning that a higher number of parental genetic backgrounds can be used, which promotes the use of heterosis. Modern rice varieties are basically semi-dwarf cultivars, intrinsically carrying a recessive dwarf gene. The overwhelming majority of rice dwarf mutants are generated from rice varieties already containing a dwarf gene, which makes it difficult to evaluate the dwarfism effects of new dwarf germplasms accurately for additive effects. Here, we evaluated the dwarfism effects of *Slr1-d6* in the genetic background of 9311 with *sd1* eliminated. In the same genetic background of 9311, *Slr1-d6* showed stronger dwarfism ability than *sd1*. The production of F_1_ hybrid seeds requires sterile parents that are 10–20 cm shorter than the pollen donor parents to increase pollen shedding on the female panicle (Virmani and Edwards, 1983).

Here we provide strong evidence that *Slr1-d6* is of great practical application value in rice hybrid breeding. First, the *Slr1-d6* homozygous plants, with heights of ~70 cm, are not much shorter than the main cultivated varieties of rice with plant height of between 80 cm and 120 cm (http://www.ricedata.cn/). Thus, the *Slr1-d6* homozygous plants could be used as the sterile parents, while most rice cultivars could be used as the pollen donor parents. Secondly, the seed-setting rate of *Slr1-d6* homozygous plants is ~80%, which is sufficient for production of F_1_ hybrid seeds. Moreover, the yield per plant of *Slr1-d6* heterozygous plants increased by 25% when compared with the normal 9311. Theoretically, if *Slr1-d6* is used in the sterile parents as a dominant dwarf source, the genetic backgrounds of the pollen donor parents will not be restricted, and the F_1_ hybrid plants will gain the standard *sd1* semi-dwarf plant height and have a higher yield potential for carrying the heterozygous genotype of *Slr1-d6*. The *Slr1-d6* heterozygous plants that have been selected for yield testing had less competition pressure for space from their neighbors in the segregation population, which would contribute to their yield just like a marginal effect. However, the exact yield-increasing potential of *Slr1-d6* in heterosis utilization still needs to be fully evaluated with more cross-combinations.

In rice, mutants of SLR1 located in DELLA/TYHYNP and SAW subdomains may lead to dominant dwarf phenotypes (Asano *et al.*, 2009; Hirano *et al.*, 2010; Zhang *et al.*, 2016). In addition, overexpression of *SLR1* variants that are mutated in LHRI, VHIID, LHRII, and PFYRE subdomains could also generate dwarfing transgenic seedlings (Hirano *et al.*, 2012). These studies indicate that mutation of SLR1 in multiple subdomains could lead to the generation of dominant dwarf mutants, making *SLR1* the prime target gene for generating dominant dwarf sources. In light of great yield-increasing potential of *Slr1-d6* and gene-editing techniques developed in rice (Shan *et al.*, 2014; Hu *et al.*, 2016), we propose that the rice DELLA protein-encoding gene, *SLR1*, is a potential target for gene editing in rice molecular breeding.

## Supplementary data

Supplementary data are available at *JXB* online.

Fig. S1. Gross morphology of transgenic seedlings and mature plants of ZX3037^*SLR1*^ and ZX3037^*Slr1-d6*^.

Table S1. Plant height and internode length comparison between ZX3037 and *Slr1-d6.*

Table S2. List of the PCR-based molecular markers developed for gene mapping.

Table S3. Primers used for sequencing identification, qRT-PCR, and yeast two-hybrid assay.

Table S4. Statistics of agronomic traits of different 9311 lines.

Table S5. Agronomic trait performance of the *Slr1-d6* homozygotes and heterozygote compared with 9311.

## Supplementary Material

Supplementary Figure S1 and Table S1-s5
