# Supplementary material for: Characterization of a new semi-dominant dwarf allele of SLR1 and its potential application in hybrid rice breeding
Source: J Exp Bot. 2018 Jun 28;69(20):4703–13. doi: 10.1093/jxb/ery243 (PMC6137977; doi:10.1093/jxb/ery243)
Supplement: Supplementary Figure S1 and Table S1-s5 [file ery243_suppl_supplementary_figure_s1_table_s1_s5.pdf]

Characterization of a new semi-dominant dwarf allele of SLR1 and its potential application in hybrid rice breeding

Zhukuan Cheng , Zhigang Wu, Ding Tang, Kai Liu, Xiaoxuan Zhuo, Chunbo Miao, Yafei Li, Mingfa Sun, Xuelin Tan, and Qiong Luo

Supplemental Data

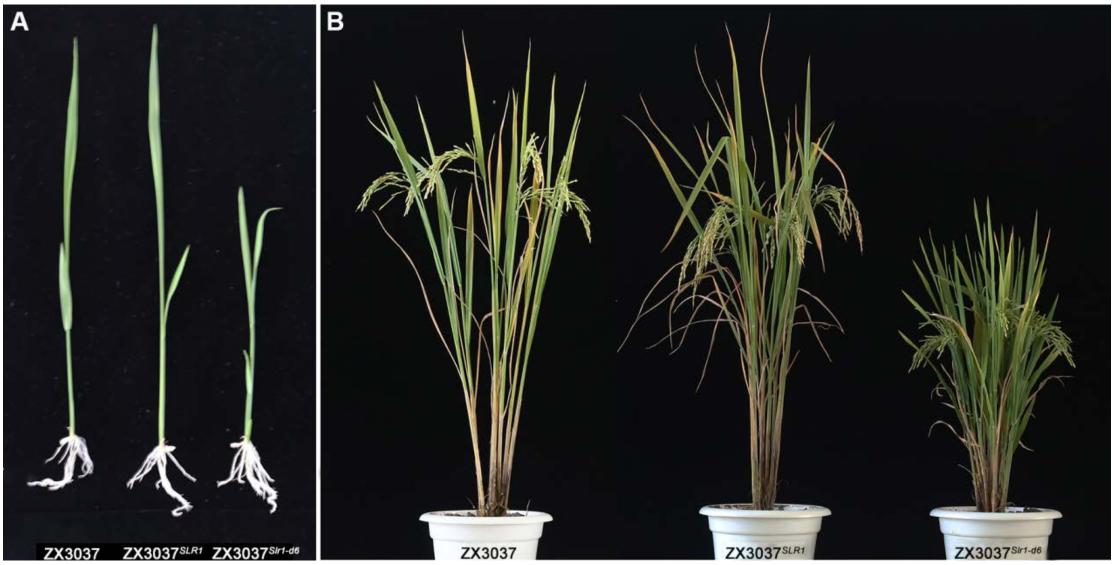

Supplementary Fig. S1. Gross morphology of transgenic seedlings and mature plants of ZX3037^{SLR1} and ZX3037$^{Slr1\text{-}d6}$. **(A)** Ten-days-old seedlings of ZX3037 (left), ZX3037^{SLR1} (middle) and ZX3037$^{Slr1\text{-}d6}$ (right). **(B)** Mature plants of wild type ZX3037 (left), ZX3037^{SLR1} (middle) and ZX3037$^{Slr1\text{-}d6}$ (right).

Supplementary Tables

Supplemental Table S1 Plant height and internode length comparison between ZX3037 and *Slr1-d6*

Name	ZX3037	*Slr1-d6*	Shortening degree
Plant height (cm)	81.84 ± 4.73	50.85 ± 2.49	37.53 %
Panicle (cm)	24.18 ± 1.71	19.91 ± 0.85	17.66 %
1st (cm)	29.40 ± 1.73	16.38 ± 0.81	44.29 %
2nd (cm)	12.87 ± 1.18	6.76 ± 0.89	47.47 %
3rd (cm)	7.78 ± 1.15	4.86 ± 0.91	37.53 %
4th (cm)	5.23 ± 0.80	2.00 ± 0.45	61.76 %
5th (cm)	2.39 ± 0.64	0.93 ± 0.16	61.09 %

*Annotation: n = 15

Supplementary Table S2. List of the PCR-based molecular markers developed for gene mapping

Markers	Primer sequence	PCR fragment length（bp）	BAC NO.
C3S10	F, 5’-TGTTGCTGCTGCTGCTTTCT-3’;<br>R, 5’-GGATAGGCCACCATTAATCA-3’	J=131<br>I=147	AC135792
C3S12	F, 5’-TTGTCCGGCATCATGGATTC-3’;<br>R, 5’-CATCCACCATTTGATGGCCT-3’	J=123<br>I=105	AC103550
P1	F, 5’-TCCAGGAAACATCCATCTCA-3’;<br>R, 5’-GACTTAATCTGTACTCGTCT-3’	J=147<br>I=177	AC084406
P2	F, 5’-CAATATGGTGCTAGGCGGAT-3’;<br>R, 5’-AATAACCTGAGCAGGCTAGG-3’	J=139<br>I=156	AC123974
P3	F, 5’-CGTTCCGCTTGGCTTTCC-3’;<br>R, 5’-CATCCTCGCAGATCACAAGT-3’	J=118<br>I=134	AC093017
P4	F, 5’-TGGAATATGCATCTAGTGAGTTG-3’;<br>R, 5’-ATTTCCTGATGAGTGGTGGT-3’	J=146<br>I=126	AC091775
P5	F, 5’-CATGAAAGAAGATTCTACCATTG-3’;<br>R, 5’-CTTGTGGTACATAAGTCTGAAAC-3’	J=143<br>I=117	AC082645
P6	F, 5’-AACCTTCTAGCATTGCACAT-3’;<br>R, 5’-GGAAGAAAGCTACTACCAC-3’	J=136<br>I=118	AC090882
P7	F, 5’-TTGGGTATCTGATCTATGGT-3’;<br>R, 5’-CAGATGCGAATTCAATACTC-3’	J=166<br>I=190	AC147426
P8	F, 5’-CTACTGCTACAGAGCCAATA-3’;<br>R, 5’-ATACCCTCAATGTCCAGTGG-3’	J=126<br>I=109	AC135956

*Annotation: J means *japonica* rice, while I represent *indica* rice.

Supplementary Table S3. Primers used for sequencing identification, qRT-PCR and yeast two-hybrid assay.

Primer name	Primer sequence
SLR1-SI-F	5’-ATCCCAAAGCCGAAACCGAG-3’
SLR1-SI-R	5’-TCGAGGATGGCTTGATTTGC-3’
SD1-SI-F	5’-ACCATCATGTCTGTCCAGTG-3’
SD1-SI-R	5’-GAACTCCCTGTAGTAGCCTC-3’
SLR1-RT-F	5’-GACCAGGTCATGTCCGAGGTG-3’
SLR1-RT-R	5’-GTAGGCATTGGAGCCCAGGTG-3’
OsCPS1-RT-F	5’-TCATGCCAAGATGTTACTTTCACAG-3’
OsCPS1-RT-R	5’-CACAATGCTCAAGAAGGTCTGCT-3’
OsKS1-RT-F	5’-AGCACTTGTGGGCGTCTCCT-3’
OsKS1-RT-R	5’-CCCTGTTCTCCAAGGACCAATCT-3’
OsKAO-RT-F	5’-GCAGAACGGATTAACCCTCAG-3’
OsKAO-RT-R	5’-GGGGATAAGATAACCGTTCACA-3’
OsGA3ox2-RT-F	5’-CCACATCCTCACCAACGGC-3’
OsGA3ox2-RT- R	5’-GAGGAAGTAGCCGAGCGAGAC-3’
SD1-RT-F	5’-ACAGGAAGAACGCCAGCGAC-3’
SD1-RT- R	5’-TGGTCATCAACATCGGCGAC-3’
OsActin-RT-F	5’-GTGGTCGCCCCTCCTGAAAG-3’
OsActin-RT-R	5’-GGCTTAGCATTCTTGGGTCCG-3’
SLR1-AD-F	5’-TGGCCATGGAGGCCAGTATGGCGGAGGGTGTAGTGG-3’
SLR1-AD-R	5’-GATGCCCACCCGGGTGTTGGATCCTTTCTGCAGCCA-3’
GID1-BD-F	5’-ATATGGCCATGGAGGCCATGGCGGAGGGTGTAGTGG-3’
GID1-BD-R	5’-GTCGACGGATCCCCGGTTGGATCCTTTCTGCAGCCA-3’

Supplementary Table S4. Statistics of agronomic traits of different 9311 lines

**Genotype**	***SD1SD1/ SLR1SLR1***	***sd1sd1/ SLR1SLR1***	***SD1SD1/ Slr1-d6SLR1***	***SD1SD1/ Slr1-d6Slr1-d6***
Plant height (cm)	165.85 ± 7.07	104.60 ± 4.19	102.18 ± 2.62	68.01 ± 2.69
Effective tillers per plant	5.36 ± 1.09	7.03 ± 1.14	8.70 ± 1.06	10.04 ± 1.22
Spikelets per panicle	251.10 ± 24.65	205.30 ± 12.66	214.80 ± 14.59	176.50 ± 15.91
Seed setting (%)	90.33 ± 3.91	87.06 ± 5.17	87.65 ± 2.96	79.08 ± 5.17
Kilo-grain weight (g)	31.30 ± 0.02	31.37 ± 0.03	31.33 ± 0.02	30.89 ± 0.04
Yield per plant (g)	28.80 ± 6.56	31.75 ± 6.67	39.95 ± 6.59	28.35 ± 5.70
Yield per m^2 (g)	946.67 ± 83.12	1086.67 ± 54.85	—	900.00 ± 37.75

Supplementary Table S5. Agronomic traits performance of *Slr1-d6* hemozygote and heterozygote compared with 9311.

Agronomic traits	***Slr1-d6* homozygote VS 9311**		***Slr1-d6* heterozygote VS 9311**	
	**Increased number**	**Increased degree**	**Increased number**	**Increased degree**
Plant height (cm)	-36.59	-34.98%	-2.42	-2.31%
Effective tillers per plant	3.01	42.82%	1.67	23.76%
Spikelets per panicle	-28.80	-14.03%	9.50	4.63%
Seed setting (%)	-7.98	-9.17%	0.59	0.68%
Kilo-grain weight (g)	-0.48	-1.53%	-0.04	-0.13%
Yield per plant (g)	-3.40	-10.71%	8.2	25.83%
Yield per m^2 (g)	-186.67	-17.18%	—	—

*Annotation: *Slr1-d6* hemozygote and heterozygote with the genetic background of 9311.
